# Decreased Expression of GATA2 Promoted Proliferation, Migration and Invasion of HepG2 In Vitro and Correlated with Poor Prognosis of Hepatocellular Carcinoma

**DOI:** 10.1371/journal.pone.0087505

**Published:** 2014-01-30

**Authors:** Yi-Wei Li, Jia-Xing Wang, Xin Yin, Shuang-Jian Qiu, Han Wu, Rui Liao, Yong Yi, Yong-Sheng Xiao, Jian Zhou, Bo-Heng Zhang, Jia Fan

**Affiliations:** 1 Liver Cancer Institute, Zhongshan Hospital, Fudan University, Key Laboratory of Carcinogenesis and Cancer Invasion (Fudan University), Ministry of Education, Shanghai, China; 2 Biomedical Research Center, Zhongshan Hospital, Fudan University, Shanghai, China; 3 Institute of Biomedical Sciences, Fudan University, Shanghai, China; Xiangya Hospital of Central South University, China

## Abstract

**Background:**

GATA family of transcription factors are critical for organ development and associated with progression of various cancer types. However, their expression patterns and prognostic values for hepatocellular carcinoma (HCC) are still largely unknown.

**Methods:**

Expression of GATA transcription factors in HCC cell lines and tissues (n = 240) were evaluated by RT-qPCR, western blot and immunohistochemistry. Cellular proliferation, migration and invasion of HepG2 was evaluated by CCK-8 kit, scratch wound assay and transwell matrigel invasion assay, respectively.

**Results:**

GATA2 expression was decreased in HCC cell lines (p = 0.056 for mRNA, p = 0.040 for protein) and tissues (p = 1.27E-25) compared with normal hepatocytes. Decreased expression of intratumoral GATA2 protein significantly correlated with elevated alpha feto-protein (p = 2.7E-05), tumor size >5 cm (p = 0.049), absence of tumor capsule (p = 0.002), poor differentiation (p = 0.005), presence of tumor thrombi (p = 0.005) and advanced TNM stage (p = 0.001) and was associated with increased recurrence rate and decreased overall survival rate by univariate (p = 1.6E-04 for TTR, p = 1.7E-04 for OS) and multivariate analyses (HR = 0.63, 95% CI = 0.43–0.90, p = 0.012 for TTR; HR = 0.67, 95% CI = 0.47–0.95, p = 0.026 for OS). RNAi-mediated knockdown of GATA2 expression significantly enhanced proliferation, migration and invasion of HepG2 cell in vitro.

**Conclusions:**

Decreased expression of hematopoietic factor GATA2 was associated with poor prognosis of HCC following resection.

## Introduction

Liver cancer is the fifth (seventh) most common cancer and the second (sixth) leading cause of cancer death in men (women) worldwide [1]. Half of these cases and deaths were estimated to occur in China [2]. Despite the great advancement in treatment modalities, especially molecular targeted therapies (e.g. Sorafenib), the outcome remains poor due to frequent recurrence [3]. It’s therefore of great importance to seek optimal biomarkers predicting tumor recurrence.

The GATA family of transcription factors, consisting of conserved proteins that contain one or two C2–C2-type zinc-finger motifs that recognize the consensus DNA sequence A/T-GATA-A/G, play crucial roles in organ development and lineage specification [4]. There are six members of mammalian GATA families, which have distinct and restricted tissue expression patterns. GATA1 and GATA2 are mainly thought as hematopoietic factors and intensely studied in hematopoietic malignancies [5]–[7]. GATA3 has been widely accepted as a classical modulator of T helper type 2 (Th2) immune response [8], which is reported to promote progression of breast [9] and pancreatic cancers [10]. Meanwhile, GATA3 has been demonstrated as a tumor suppressor gene of breast tumor [11]. GATA4, GATA5 and GATA6 are mainly thought as endodermal factors and proven to be differentially expressed in normal and malignant tissues of endodermal origin [12]. Loss of function of GATA4 ∼ 6 by promoter hypermethylation [13]–[15] or nucleocytoplasmic mislocalization [16], [17] is a common event in carcinoma of lung, digestive tract, pancrea and ovarian, which causes loss of expression of epithelial-specific markers (disabled-2, collagen IV and laminin) leading to cellular dedifferentiation and down-regulates potential targets of tumor suppressor genes (the trefoil factors, inhibin and disabled-2). Targeting promoter methylation or nuclear trafficking of GATA transcription factors therefore exhibits potential antitumor effect [14], [15], [18].

Liver belongs to the organ systems of endoderm origin. In vivo footprinting study of mouse embryonic endoderm cells has demonstrated occupancy of DNA-binding site for GATA factors on a liver-specific transcriptional enhancer of the serum albumin gene [19], of which GATA4 isoform accounts for about half of the function. This phenomenon persists during hepatic development and is necessary for the activity of albumin gene enhancer. Supporting this physiological function, GATA transcription factors have been reported to be expressed in human hepatoma cell lines HepRG/HepG2/Hep3B [4] and associated with EGF-mediated induction of nucleotide excision repair activity and ERCC1 expression [20], which is an important mechanism related with platinum resistance in many cancer types [21], [22]. Nevertheless, limited information has been known about the expression pattern and prognostic influence of GATA transcription factors in human hepatocellular carcinoma. In the present study, we investigated the expression of GATA transcription factors and evaluated their prognostic importance for hepatocellular carcinoma following resection. GATA1 wasn’t further analyzed due to complete loss of expression in hepatic/HCC cell lines and tissues at both mRNA and protein levels. GATA3 was excluded due to concomitant expression at tumor and stroma cells [9], [10] as well as double-edged sword effect for HCC prognosis (unpublished data). Our results demonstrated the decreased expression of GATA2 in HCC cell lines compared with normal hepatocytes as well as in HCC tissues with recurrence compared with those without recurrence. Decreased expression of intratumoral GATA2 protein significantly correlated with pivotal clinicopathologic factors related to tumor invasiveness (elevated serum AFP level, larger tumor size, absence of tumor capsule, poor cellular differentiation, presence of tumor thrombi and advanced TNM stage) and independently predicted patient outcome. RNAi-mediated silence of GATA2 enhanced proliferation, migration and invasion of HepG2 cell in vitro. By contrast, peritumoral GATA2 or intratumoral/peritumoral GATA4 ∼ 6 showed no significant prognostic influence.

## Materials and Methods

### Patients, Follow-up and Treatment Modalities

A total of 240 pathologically confirmed hepatocellular carcinoma patients, who received curative resection in Liver Cancer Institute, Zhongshan Hospital, Fudan University between January 2002 and December 2006, were enrolled in this study. The inclusion criteria were: 1) with good performance status and compensated liver function, 2) without evident metastasis to distant organs before surgery, 3) without preoperative anticancer therapy, 4) without residual cancer in the liver remnant. This study was approved by Zhongshan Hospital Research Ethics Committee and written informed consent was obtained from all the patients.

All the patients received standardized follow-up established by our institute [23], [24]. Primary endpoints were time to recurrence (TTR) and overall survival (OS) defined as time intervals between the date of surgery and first report of tumor recurrence or patient death, respectively. Patients without recurrence or death were censored. The follow-up was completed on Mar 31^st^ 2011, with a median follow-up of 44.4 months (range 2.0∼106.8 months). Conventional clinicopathologic data and treatment modalities were detailed in **Supplementary Table S1**.

### Tissue Microarray and Immunohistochemistry

Tissue microarrays were constructed as described previously [23]. Briefly, representative areas, away from necrotic and hemorrhagic areas according to H&E staining, were premarked on the paraffin blocks. Triplicates of 1 mm diameter core were taken from tumor center and noncancerous margin in each case (designated as intratumor and peritumor, respectively) to ensure reproducibility and homogeneity.

Immunohistochemistry was carried out according to appropriate protocols described in our previous reports and elsewhere [23], [24]. Briefly, after deparaffinization, hydration and blocking of endogenous peroxidase (0.3% H_2_O_2_ for 20 min), antigen retrieval was performed in pH 8.0 Tris-EDTA using a microwave oven for 15 min. Sections were then incubated with 5% bovine serum albumin (BSA, Sigma-Aldrich, Inc) at room temperature for 30 min and primary antibodies (GATA1 1:100, Cell Signaling Technology 3535; GATA2 1:800, Abcam ab22849; GATA4 1:500, Santa Cruz sc-25310; GATA5 1:500, Sigma-Aldrich G8669; GATA6 1:100, R&D AF1700) at 4°C overnight. Slides were then applied in the detection system of Elivision™ Plus Kit and DAB following counterstaining with hematoxylin. The slides were washed in pH 7.4 TBS after every step but not after incubation with 5% BSA. Primary antibodies were replaced by TBS as blank control and breast tumor tissue was applied as positive control.

Images of immunohistochemistry were recorded at 40× and 200× magnification of light microscopy, which were then digitalized and analyzed using Image-Pro Plus 6.0 software (Media Cybernetics Inc, Bethesda, MD) as previously described [25]. The staining intensity was expressed as average density of triplicate cores. The results were confirmed by two experienced pathologists who were blinded to clinicopathologic data of patients.

### Cell Culture, Protein Extraction and Western Blot

Human HCC cell lines MHCC97-L, MHCC97-H and HCCLM3 were established in our institute as previously described [26]. Human HCC cell lines (Hep G2, BEL-7402, Huh-7, SMMC7721) and human hepatic cell lines (normal hepatocyte: HL-7702[L-02]; peritumoral hepatocyte: QSG-7701) were purchased from the Type Culture Collection of the Chinese Academy of Sciences, Shanghai, China. All the cell lines were cultured in high glucose DMEM or RPMI 1640 medium as appropriate and supplemented with 10% fetal bovine serum (Gibco, NY) in a humidified atmosphere of 5% CO_2_ at 37°C.

Western blot was performed as described previously and elsewhere [27]. Briefly, cell or tissue lysates were generated and total proteins were separated by standard SDS-PAGE, followed by transfer to polyvinylidene difluoride membranes. The membranes were then washed and blocked before incubation of primary antibodies (GATA1 1:200, GATA2 1:500, GATA4 1:200, GATA5 1:500, GATA6 1:250, GAPDH 1∶5000), followed by incubation of HRP-conjugated secondary antibodies. The reactions were detected by enhanced chemiluminescence assay. GAPDH was used as a loading control. The relative intensity of each band was determined by Image Lab 3.0 (Bio-Rad Laboratories, Inc).

### RNA Preparation and Quantitative Real-time PCR

Total RNA was extracted using the RNeasy Mini Kit (Qiagen, Valencia, CA). Quantitative Real-time PCR was performed according to the manufacturer’s instructions (the Quant SYBR Green PCR Kit, Tiangen Biotech, Beijing). ACTB (beta-actin) and HPRT1 (hypoxanthine phosphoribosyltransferase 1) were used as loading controls for cell lines and tissues, respectively. The conditions were as follows: 10 min at 95°C, followed by 40 cycles of 95°C for 15 sec and 60°C for 60 sec. The primers were shown at **Supplementary Table S2**. ACTB and HPRT1 were used as loading control. The relative quantification was calculated by 2^−ΔCt^ method (ΔCt = Ct^[target]^ − Ct^[HKG]^) for cell lines or 2^−ΔΔCt^ method [28] (−ΔΔCt = ΔCt^[normal]^ −ΔCt^[tumor]^) for tissues.

### RNAi-mediated GATA2 Silencing

Expression of GATA2 was silenced with siRNA sequence (sense 5′ UUCUUGGACUUGUUGGACAUCUUCC-3′, antisense 5′-GGAAGAUGUCCAACAAGUCCAAGAA-3′) reported elsewhere [29] and designed by Shanghai GenePharma Co. Transfection of the siRNAs for HepG2 cell was performed with Lipofectamine 2000 (Invitrogen, Carlsbad, CA) according to the manufacturer’s instructions. After 72 hours of transfection, cells were lysed for western blot analysis.

### Cell Proliferation, Migration and Invasion Assay

### Cell Proliferation Assay

Cell proliferation was determined by Cell Counting Kit 8 (CCK-8) as described previously [30]. Briefly, 10^3^ cells/well of HepG2 in 100 µl aliquots was dispensed into 96-well plate. At timepoints of day 0, day 2, day 4 and day 6, CCK-8 was added to the wells and incubated for 1 hour, and then the absorbance at 450 nm was calculated.

### Cell Migration Assay

Cell migration was evaluated by scratch wound assay [31]. Briefly, 10^6^ cells/well of HepG2 were plated in 6-well plate and cultured overnight to yield confluent monolayer. Cells were treated with 10 µg/ml mitomycin for 1 hour to inhibit proliferation [32], followed by wounding with 10 µl pipette tip. Remaining cells were washed twice and then cultured with DMEM supplemented with 2% FBS. Photographs were taken at timepoints of 0 hour, 24 hours, 48 hours and 72 hours.

### Cell Invasion Assay

Cell invasion was evaluated by transwell matrigel invasion assay [33]. 10^5^ cells/well of HepG2 suspended with DMEM were seeded in the upper chamber coated with matrigel and incubated with DMEM supplemented with 10% FBS and HepG2 conditioned supernatant in the lower chamber. After 48 hours, invaded cells on the bottom surface were fixed with 4% paraformaldehyde and quantified after staining with crystal violet.

### Statistic Analysis

Comparison among different groups was analyzed using paired samples t tests or Mann-Whitney U tests as appropriate. Correlations between different variables were determined by Spearman coefficient t tests. Univariate and multivariate analysis were done by Kaplan-Meier method and Cox proportional hazards regression model and compared by the log-rank test. The “minimum p value” approach [34] was used to get optimal cut-off for the best separation of patients’ TTR. For each analysis, only p<0.05 (two-sided) was considered statistically significant. All statistic analyses were made by SPSS 17.0 (SPSS Inc, Chicago, IL, USA).

## Results

### Loss of Expression of Hematopoietic Factor GATA1 in Hepatic and HCC Cell Lines and Tissues

**Figure 1:**
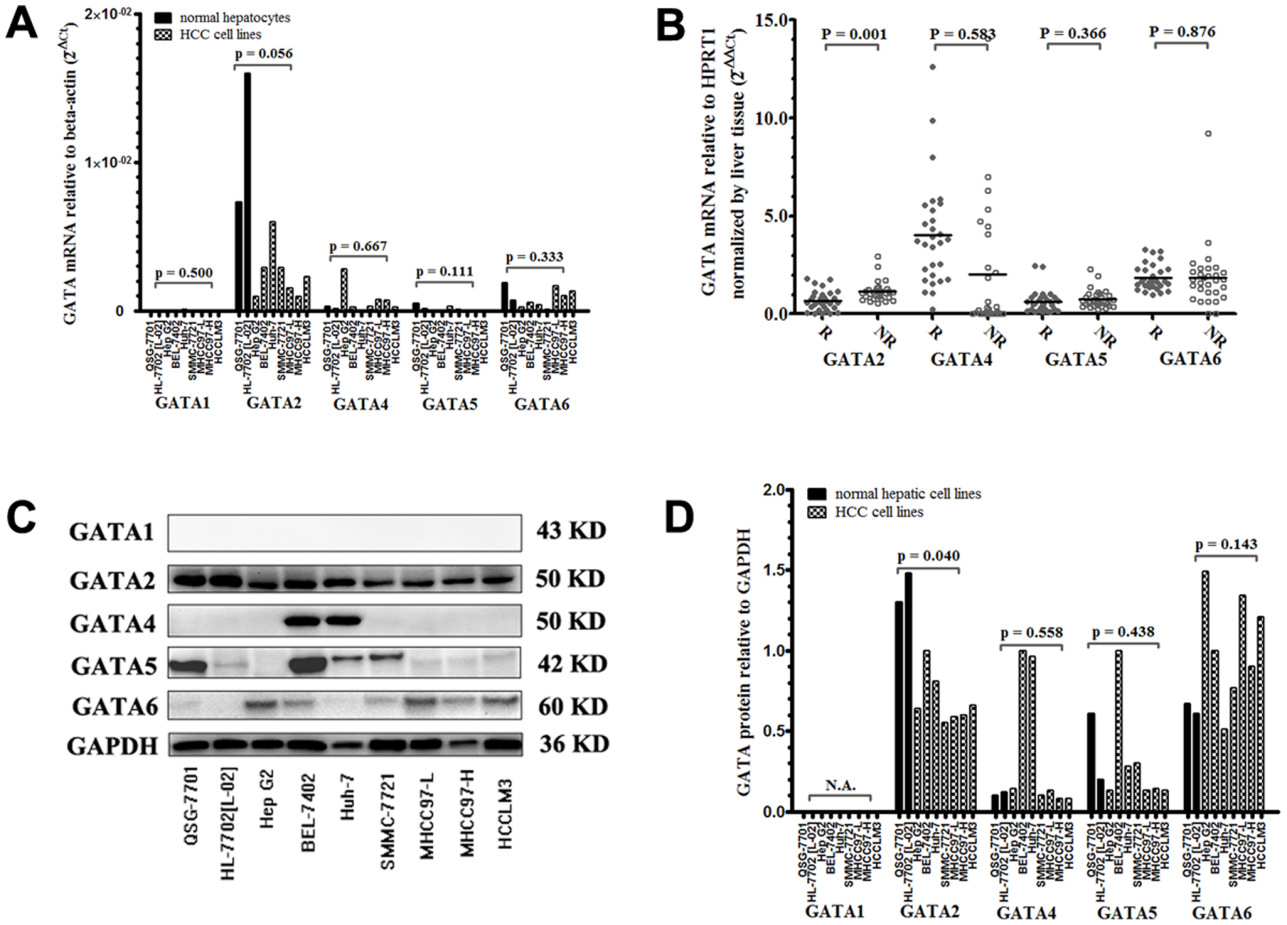
Expression of GATA transcription factors in hepatic/HCC cell lines (A, C, D) and tissues (B). Expression of GATA2 mRNA (A, p = 0.056) and protein (D, p = 0.040) was decreased in HCC cell lines compared with hepatocytes (QSG-7701 and L-02). Decreased expression of GATA2 mRNA in tumor tissues was significantly associated with tumor recurrence (B, p = 0.001). Experiments of cell lines were repeated at least three times. Mann-Whitney U tests. R: recurrence, NR: non-recurrence, N.A.: not applicable.

We showed by RT-qPCR that GATA1 mRNA was expressed at relatively low level in majorities of hepatic and HCC cell lines analyzed (2^−△Ct^, mean 1.1E-05, range 6.6E-07∼6.9E-05, approximately 1000 times less than other GATAs’ expression, **Fig. 1A**). Western blot and immunohistochemistry confirmed complete loss of expression of GATA1 protein in hepatic and HCC cell lines (**Fig. 1C–D**) as well as in 80 HCC tissues from one section of tissue microarray (**Supplementary** **Fig. S1A**). Therefore, GATA1 was no longer evaluated in the latter experiments.

### Ectopic Expression of Hematopoietic Factor GATA2 in Hepatic and HCC Cell Lines and Tissues

GATA2 mRNA was abundantly expressed in normal hepatocytes and trended to be down-regulated in HCC cell lines (mean 0.011 vs 0.002, p = 0.056, **Fig. 1A**). Western blot confirmed abundant expression of GATA2 protein in all the cell lines as well as decreased expression in HCC cell lines compared with normal hepatocytes (p = 0.040, **Fig. 1C–D**). Expression of GATA2 mRNA was significantly elevated in 30 HCC tissues without recurrence compared with 30 HCC tissues with recurrence (2^−△△Ct^, mean 0.693 vs 1.146, p = 0.001, **Fig. 1B**).

### Frequent Loss of Expression of Endoderm Factors GATA4, GATA5 and GATA6 in Hepatic and HCC Cell Lines and Tissues

Expression of GATA4 and GATA5 was frequently lost while GATA6 was partially maintained at both mRNA (2^−△Ct^, mean 0.0006, 0.0001 and 0.0009, respectively, **Fig. 1A**) and protein levels (7/9, 5/9 and 2/9 negative, **Fig. 1C**) in hepatic and HCC cell lines. However, no significant difference was found with respect to their expression between normal hepatocytes and HCC cell lines (mRNA/protein: GATA4, p = 0.667/0.558; GATA5, p = 0.111/0.438; GATA6, p = 0.333/0.143; respectively, **Fig. 1A and D**). In 60 HCC tissues with recurrence or not, expression of GATA5 mRNA was 3 folds down-regulated while GATA4 and GATA6 mRNA was 3 times and 2 times up-regulated compared with normal liver tissues. No significant difference was found with respect to their expression in HCC tissues with recurrence or not (p = 0.583, 0.336 and 0.876 for GATA4, GATA5 and GATA6, respectively, **Fig. 1B**).

### Expression Pattern of GATA Proteins in Paired Tumor and Peritumor Tissues and Correlation with Clinicopathologic Factors

**Figure 2:**
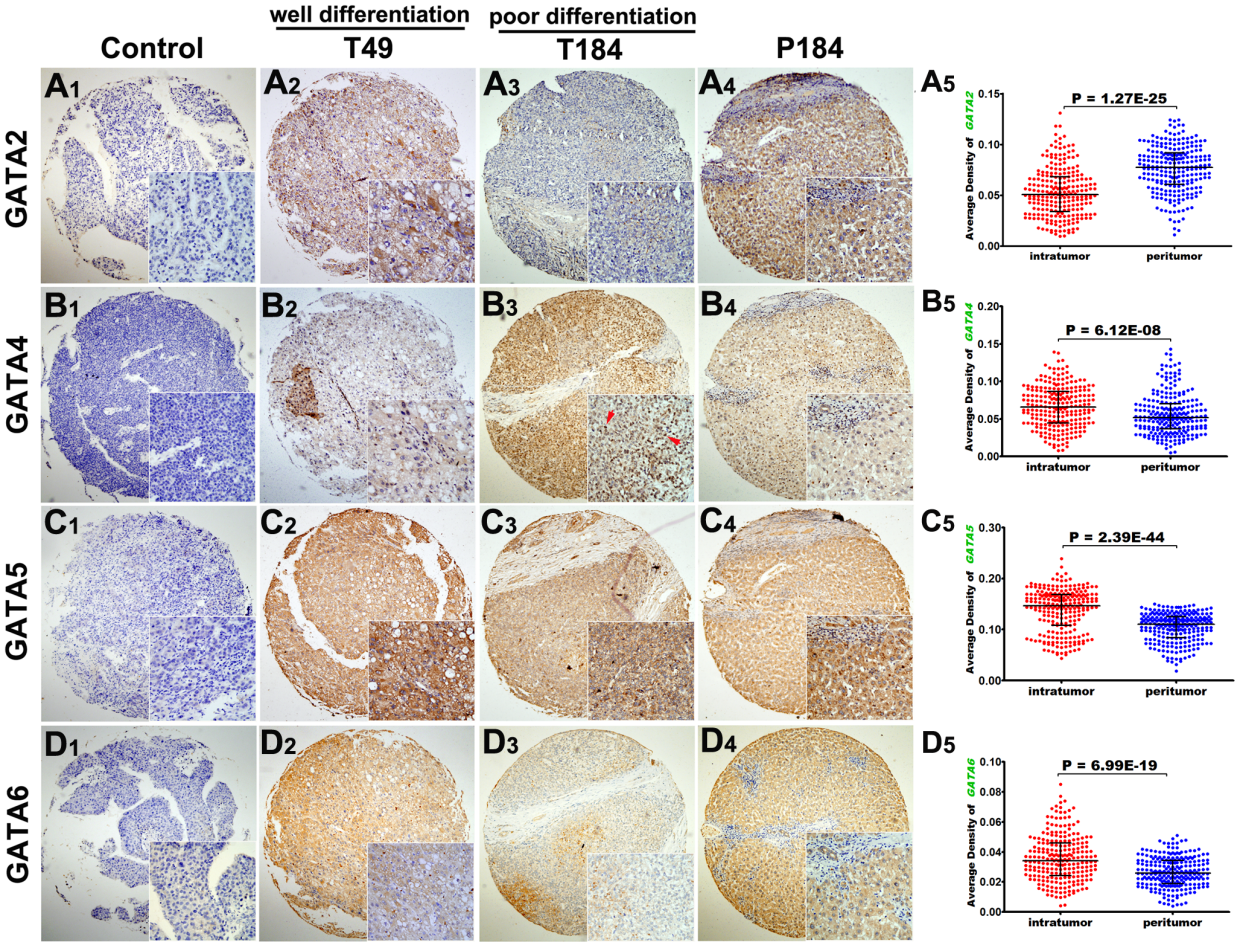
Representative images and quantitative density of GATAs in paired tumor and peritumor tissues from consecutive sections of tissue microarray (40× and 200×). Expression of GATA2 (**A_5_**) protein was decreased, GATA4 (**B_5_**), GATA5 (**C_5_**) and GATA6 (**D_5_**) was increased in tumor tissues compared with adjacent liver tissues. T49/T184 represented tumor tissues with well/poor differentiation state. P184 was the corresponding peritumor tissue of T184. Paired samples t tests for A5, B5, C5 and D5.

We determined the expression pattern of GATA binding proteins in paired tumor and peritumor tissues of 240 patients undergoing curative resection. Except that some tumor tissues exhibited nuclear positivity of GATA4 (**Fig. 2B** **3, T184, red arrow**), majorities of intratumoral (**Fig. 2** **, T49&T184**) and peritumoral (**Fig. 2** **, P184**) tissues showed diffuse cytoplasmic expression pattern of GATA proteins. The proportion of cases showed negative expression, poor positivity and strong positivity of intratumoral or peritumoral GATAs were shown in **Supplementary Fig. S1**.

We then evaluated the relative expression level of GATAs protein in tumor and peritumor of tissue microarray by average density of immunostaining using Image-Pro Plus 6.0 software (Media Cybernetics Inc, Bethesda, MD) [35]. As was shown in **Supplementary Fig. S1B–E**, among negative, poor positive and strong positive groups, the average densities were significantly different for intratumoral GATA2 (p = 3.4E-34 and 2.9E-07), GATA4 (p = 3.4E-11 and 3.6E-27), GATA5 (p = 1.5E-05 and 1.7E-21), GATA6 (p = 2.0E-16 and 0.001) as well as for peritumoral GATA2 (p = 8.0E-19 and 1.4E-19), GATA4 (p = 2.0E-15 and 2.6E-19), GATA5 (p = 1.4E-07 and 7.5E-42) and GATA6 (p = 1.6E-10 and 1.1E-11). The consistency indicated high reliability and reproducibility of this method. Compared with tumor tissues, paired peritumor tissues had significantly elevated expression of GATA2 (mean, 0.050 vs 0.077, p = 1.27E-25, **Fig. 2A** **5**) and decreased expression of GATA4 (mean, 0.065 vs 0.051, p = 6.12E-08, **Fig. 2B** **5**), GATA5 (mean, 0.014 vs 0.010, p = 2.39E-44, **Fig. 2C** **5**) and GATA6 (mean, 0.036 vs 0.027, p = 6.99E-19, **Fig. 2D** **5**).

### Determination of Optimal Cut-offs by ‘Minimum p value Approach’

**Figure 3:**
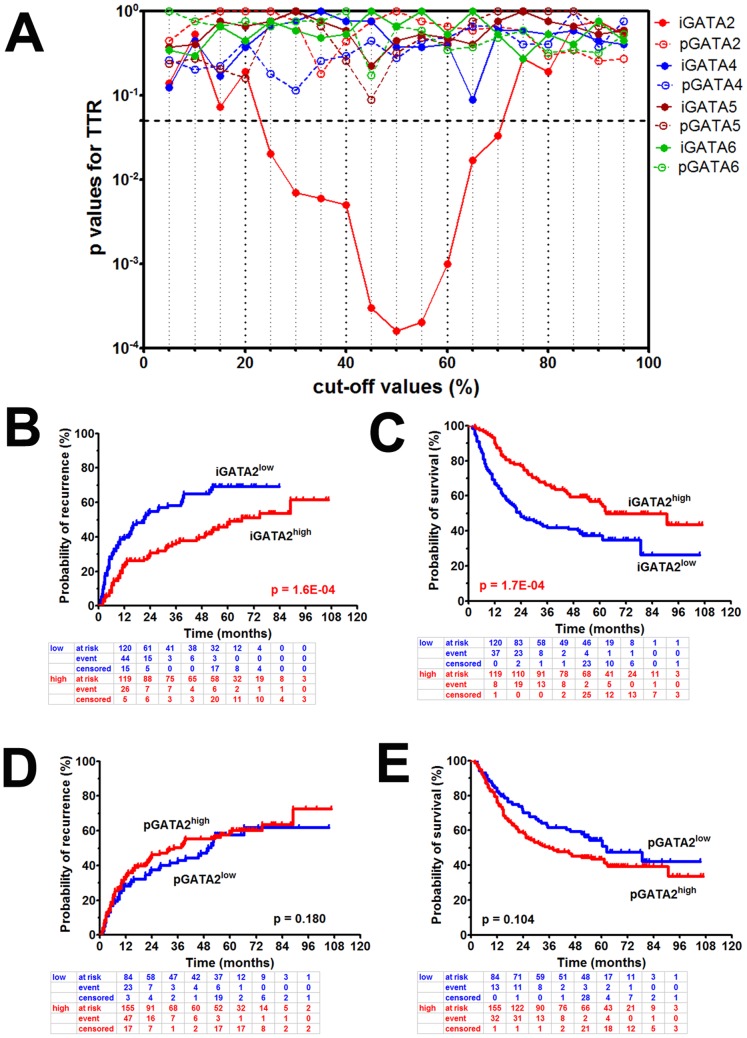
Minimum p values of GATA proteins (A) for recurrence and Kaplan-Meier analyses of GATA2 for recurrence (B, D) and death (C, E). Cut-off values of intratumoral GATA2 reaching statistical significance ranged from 25% to 70% (**A**, dashed line indicated p = 0.05). Decreased intratumoral GATA2 expression (blue line) significantly correlated with increased recurrence rate (**B**) and decreased overall survival (**C**). Frequencies of patients at risk, event or censored were created by life table method. High and low subgroups were defined by optimal cut-off using minimum p value approach.

For better prediction of patient outcome and optimal seperation of subgroups determined by GATAs immunostaining, we used ‘minimum p value approach’ [34]–[36]. As was shown in **Fig. 3A**, the significant cut-offs of intratumoral GATA2 expression for recurrence-free survival ranged from 25^th^ percentile to 70^th^ percentile (p = 1.6E-04∼0.033, with minimum p value at 50^th^ percentile), indicating high efficiency and good reproducibility predicting tumor recurrence. However, no significant cut-offs were achieved for peritumoral GATA2 (p_min_ = 0.180 at 35^th^ percentile) or intratumoral/peritumoral GATA4 (p_min_ = 0.089 at 65^th^ percentile; p_min_ = 0.114 at 30^th^ percentile), GATA5 (p_min_ = 0.221 at 45^th^ percentile; p_min_ = 0.088 at 45^th^ percentile) and GATA6 (p_min_ = 0.273 at 75^th^ percentile; p_min_ = 0.173 at 45^th^ percentile).

### Correlation of GATAs Expression with Clinicopathologic Factors

Using cut-offs with minimum p values, we divided the cohort into low GATAs expression group and high GATAs expression group. We then evaluated the correlation of GATAs expression with clinicopathologic factors. As was shown in **Table 1**, decreased expression of intratumoral GATA2 protein was significantly associated with female patients (p = 0.008), older age (p = 0.028), elevated pre-operative serum AFP level (p = 2.7E-05), tumor size larger than 5cm (p = 0.049), absence of tumor capsule (p = 0.002), poor cellular differentiation (p = 0.005), presence of tumor thrombi (p = 0.005) and advanced TNM stage (p = 0.001). Furthermore, peritumoral GATA2 was more abundantly expressed in male patients compared with females (p = 0.003). As was shown in **Supplementary Table S3**, decreased expression of intratumoral GATA4 protein was correlated with normal serum AFP level (p = 0.014) and well tumor differentiation (p = 0.047). However, expression of GATA5 or GATA6 proteins in tumor or peritumor tissues was not correlated with pivotal clinicopathologic factors.

**Table 1:** Correlation of GATA2 Expression with Clinicopathologic Factors.

		iGATA2		pGATA2	
		Mean Density	p	Mean Density	p
Gender	Male	0.054±0.024	0.008	0.078±0.022	0.003
	Female	0.042±0.017		0.066±0.022	
Age(Year)	≦52	0.057±0.026	0.028	0.074±0.024	0.137
	>52	0.049±0.021		0.078±0.021	
HBV infection	No	0.063±0.031	0.156	0.081±0.024	0.393
	Yes	0.052±0.023		0.076±0.023	
Liver cirrhosis	No	0.059±0.028	0.274	0.077±0.024	0.851
	Yes	0.052±0.023		0.076±0.023	
AFP(ng/ml)	≦20	0.063±0.026	2.7E-05	0.074±0.023	0.422
	>20	0.048±0.022		0.077±0.023	
ALT(U/L)	≦75	0.051±0.023	0.066	0.075±0.023	0.443
	>75	0.061±0.029		0.080±0.019	
γ-GT(U/L)	≦54	0.053±0.022	0.433	0.074±0.023	0.223
	>54	0.052±0.025		0.077±0.023	
Tumor size(cm)	≦5	0.056±0.022	0.049	0.074±0.023	0.219
	>5	0.051±0.026		0.078±0.022	
Tumor number	1	0.054±0.024	0.073	0.076±0.022	0.577
	≧2	0.049±0.025		0.074±0.024	
Tumor capsule	Yes	0.057±0.022	0.002	0.076±0.024	0.914
	No	0.048±0.025		0.077±0.021	
Differentiation	Well	0.057±0.026	0.005	0.074±0.024	0.275
	Poor	0.047±0.020		0.079±0.021	
Tumor thrombi	No	0.056±0.023	0.005	0.074±0.025	0.283
	Yes	0.049±0.024		0.078±0.020	
TNM stage	I	0.058±0.023	0.001	0.074±0.024	0.586
	II/III	0.049±0.024		0.077±0.021	
Prophylactic therapy	No	0.055±0.025	0.218	0.077±0.024	0.144
	Yes	0.050±0.023		0.074±0.022	

Note: Mann-Whitney U tests for all the analyses.

### Prognostic Significance of GATA Proteins for HCC

We showed by Kaplan-Meier analysis (**Table 2**) that decreased expression of intratumoral GATA2 protein was significantly associated with higher cumulative recurrence rate and lower overall survival rate (low vs high, 5-y: 69% vs 47%, p = 1.6E-04 for TTR; 36% vs 57%, p = 1.7E-04 for OS; **Fig. 3B–C**). However, no significant difference was found in terms of TTR (p = 0.180) and OS (p = 0.104) for the expression of peritumoral GATA2 (**Fig. 3D–E**). Besides, expression of intratumoral or peritumoral GATA4 (tumor: p = 0.089 for TTR, p = 0.138 for OS; peritumor: p = 0.114 for TTR, p = 0.381 for OS; **Supplementary Fig. S2A_1_–A_4_**), GATA5 (tumor: p = 0.221 for TTR, p = 0.281 for OS; peritumor: p = 0.088 for TTR, p = 0.174 for OS; **Supplementary Fig. S2B_1_–B_4_**) and GATA6 (tumor: p = 0.273 for TTR, p = 0.228 for OS; peritumor: p = 0.173 for TTR, p = 0.953 for OS; **Supplementary Fig. S2C_1_–C_4_**) was not significantly associated with tumor recurrence or patient death. Among those factors related to patient outcome (**Table 2**), elevated pre-operative serum AFP level (>20 ng/ml), larger tumor size (>5 cm), multiple tumor nodules, absence of tumor capsule, presence of tumor thrombi and advanced TNM stage was associated with higher cumulative recurrence rate and lower overall survival rate. Besides, presence of liver cirrhosis, elevated pre-operative γ-GT level (>54 ng/ml) and poor tumor differentiation were also associated with lower overall survival rate.

**Table 2:** Univariate and Multivariate Analyses of Prognostic Factors.

	Time to Recurrence			Overall Survival		
	Uni-	Multi-variate analysis		Uni-	Multivariate analysis	
	p	H.R. (95% CI)	p	p	H.R. (95% CI)	p
Age, year (≦52 vs >52)	0.101	N.A.	N.A.	0.097	N.A.	N.A.
Gender (female vs male)	0.281	N.A.	N.A.	0.274	N.A.	N.A.
HBV infection (no vs yes)	0.379	N.A.	N.A.	0.778	N.A.	N.A.
Liver cirrhosis (no vs yes)	0.146	N.A.	N.A.	0.038	1.54 (0.81–2.90)	0.186
ALT, U/L (≦75 vs >75)	0.695	N.A.	N.A.	0.586	N.A.	N.A.
γ-GT, U/L (≦54 vs >54)	0.121	N.A.	N.A.	3.0E-04	1.63(1.13–2.36)	0.009
AFP, ng/ml (≦20 vs >20)	0.017	1.15 (0.77–1.71)	0.483	2.1E-04	1.23 (0.81–1.88)	0.324
Tumor size, cm (≦5 vs >5)	0.001	1.12 (0.76–1.64)	0.547	7.3E-08	1.45 (0.98–2.14)	0.059
Tumor number (single vs multiple)	0.001	1.67 (1.13–2.47)	0.010	0.001	1.66 (1.13–2.45)	0.009
Tumor capsule (yes vs no)	0.021	0.96 (0.66–1.41)	0.846	0.003	0.94 (0.65–1.36)	0.758
Differentiation (well vs poor)	0.060	N.A.	N.A.	0.001	1.60 (1.13–2.26)	0.008
Tumor thrombi (no vs yes)	3.8E-11	2.91 (2.01–4.22)	1.3E-08	4.8E-18	3.58 (2.42–5.31)	1.8E-10
TNM stage (I vs II/III)	2.7E-09	N.A.	N.A.	2.3E-14	N.A.	N.A.
Mean Density (low vs high)						
iGATA2 (50%)	1.6E-04	0.63 (0.43–0.90)	0.012	1.7E-04	0.67 (0.47–0.95)	0.026
pGATA2 (35%)	0.180	N.A.	N.A.	0.104	N.A.	N.A.
iGATA4 (65%)	0.089	N.A.	N.A.	0.138	N.A.	N.A.
pGATA4 (30%)	0.114	N.A.	N.A.	0.381	N.A.	N.A.
iGATA5 (45%)	0.221	N.A.	N.A.	0.281	N.A.	N.A.
pGATA5 (45%)	0.088	N.A.	N.A.	0.174	N.A.	N.A.
iGATA6 (75%)	0.273	N.A.	N.A.	0.228	N.A.	N.A.
pGATA6 (45%)	0.173	N.A.	N.A.	0.953	N.A.	N.A.

Note: Kaplan-Meier & Cox proportional hazards regression model for univariate & multivariate analysis with log-rank test. Statistically significant variables demonstrated at univariate analysis were adopted into Cox regression model.

Variables demonstrated to be statistically significant (p<0.05) were then adopted to Cox proportional hazards regression model for multivariate analysis. Since TNM stage was a compound variable consisted of tumor size, tumor number and tumor thrombi, it was excluded from this analysis. Compared with patients with low GATA2 expression, patients with high GATA2 expression had significantly decreased risk of tumor recurrence (H.R. = 0.63, 95% CI = 0.43∼0.90, p = 0.012) and patient death (H.R. = 0.67, 95% CI = 0.47∼0.95, p = 0.026). Besides, tumor multiplicity (p = 0.010 and p = 0.009) and presence of tumor thrombi (p = 1.3E-08 and p = 1.8E-10) was associated with elevated risk of tumor recurrence and patient death; while elevated pre-operative serum γ-GT level (p = 0.009) and poor differentiation (p = 0.008) were also associated with increased risk of patient death.

A great proportion of patients (126/240, 52.5%, **Supplementary Table S1**) with high risk of tumor recurrence received prophylactic therapy after resection, which would reasonably obscure the prognostic influence of intratumoral GATA2. We found that expression of intratumoral GATA2 protein was not significantly different in subgroups of patients receiving prophylactic therapy or not (0.050±0.023 vs 0.055±0.025, p = 0.218, **Table 1**). Further analysis indicated that intratumoral GATA2 remained to be an independent predictor of tumor recurrence and patient death in subgroups of patients receiving prophylactic therapy (p = 0.039 for TTR and p = 0.005 for OS) or not (p = 0.003 for TTR and p = 0.027 for OS, **Supplementary Fig. S3**).

### GATA2 Knockdown Enhanced Proliferation, Migration and Invasion of HepG2 Cell

**Figure 4:**
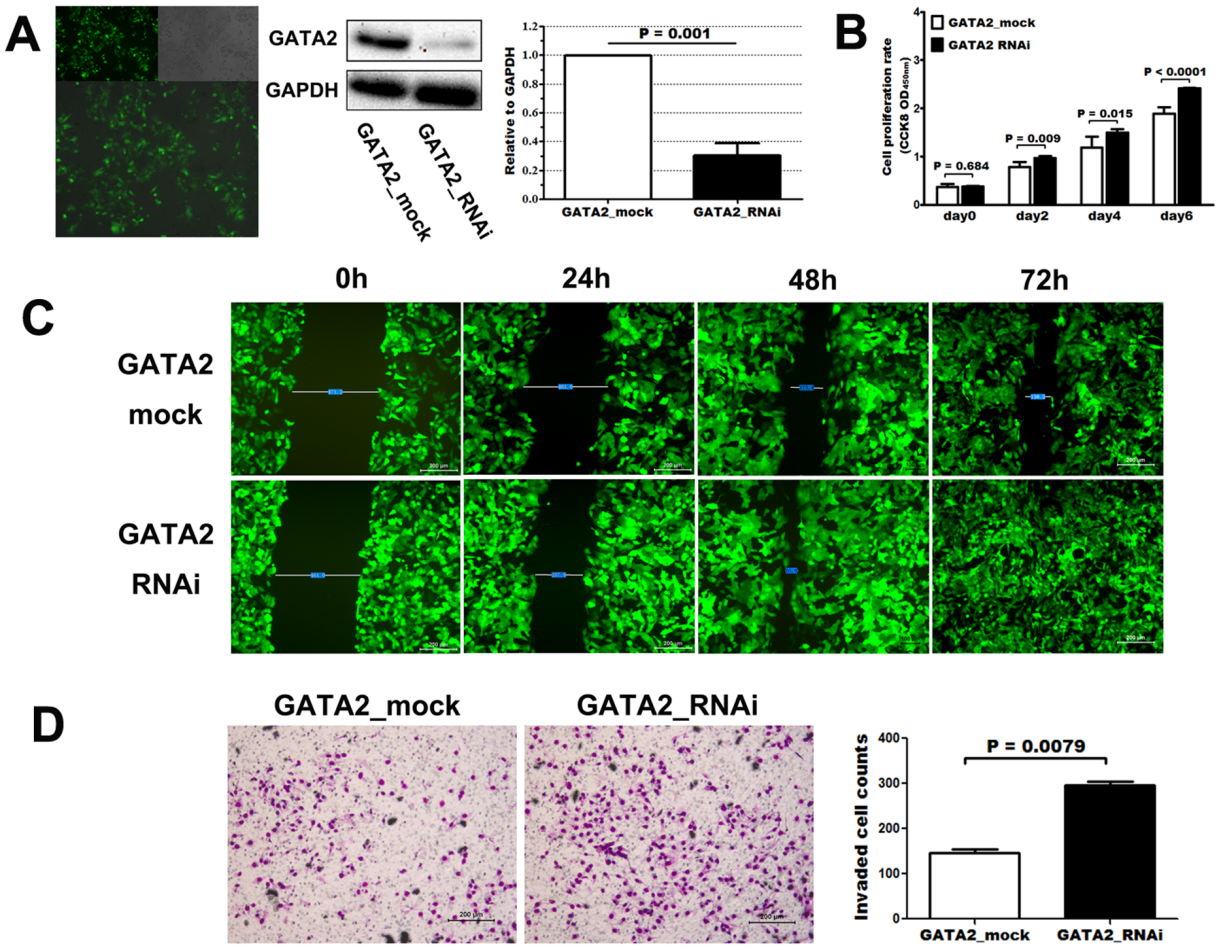
RNAi-mediated knockdown of GATA2 (A) enhanced proliferation (B), migration (C) and invasion (D) of HepG2 cell in vitro. RNAi efficiently down-regulated expression of GATA2 protein in HepG2 cell (**A**). After knock-down of GATA2 expression, cellular proliferation (**B**), migration (**C**) and invasion (**D**) of HepG2 was elevated compared with control. Mann-Whitney U tests, each experiment was repeated three times.

We used small interfering RNA (siRNA) technique to silence the expression of GATA2 in HepG2 cell. As was demonstrated by western blot analysis, GATA2-specific siRNA sequence significantly knockdown the expression of GATA2 protein in HepG2 cell (p = 0.001, **Fig. 4A**). Cell Counting Kit 8 (CCK-8) assay showed that knockdown of GATA2 expression significantly enhanced the proliferation of HepG2 cell at different timepoints (day2, day4, day6: p = 0.009, p = 0.015, p<0.0001, respectively; **Fig. 4B**) in vitro. Scratch wound assay indicated enhanced migration of HepG2 cell in siRNA group compared with control group (**Fig. 4C**). Transwell matrigel invasion assay showed that silencing of GATA2 expression significantly promoted invasion of HepG2 cell in vitro (invaded cell number, control vs siRNA: 147 vs 295, p = 0.0079, **Fig. 4D**).

## Discussion

For the first time, we investigated the expression pattern and prognostic importance of GATA transcription factors for hepatocellular carcinoma undergoing radical resection. Our results showed that GATA2 was differentially expressed in normal hepatocytes and HCC cell lines as well as in HCC tissues with recurrence or not at both mRNA and protein levels. Decreased expression of intratumoral GATA2 protein significantly correlated with pivotal clinicopathologic factors related to HCC invasiveness and independently predicted elevated risks of tumor recurrence and patient death. RNAi-mediated silencing of GATA2 expression significantly enhanced proliferation, migration and invasion of HepG2 cell in vitro.

GATA2 is mainly recognized as a modulator of early hematopoietic cell lineages and associated with progression of classical Hodgkin’s lymphoma [7] as well as other hematopoietic diseases [5]. However, accumulating evidence has uncovered its ectopic expression and potential prognostic value in solid tumors [20], [27], [37], [38]. Similarly, we have detected ectopic expression of GATA2 at both mRNA and protein levels in normal hepatocytes and HCC cell lines. Compared with normal hepatocytes, HCC cell lines exhibited decreased expression of GATA2 (**Fig. 1A and D**). Further analysis by immunohistochemistry found a tight correlation of intratumoral GATA2 protein expression with cellular differentiation (**Fig. 2**, T49 vs T184). RNAi-mediated GATA2 knockdown significantly enhanced proliferation of HepG2 cell in vitro (**Fig. 4B**). These results suggested that GATA2 might be involved in the onset and early stage of HCC progression, probably by regulating the proliferation and differentiation of HCC, which keeps in line with its role during hepatic development [19], [39]. Besides, our results also revealed a significant correlation of intratumoral GATA2 expression with other pivotal parameters related to local invasion (e.g. tumor capsule) and distant metastasis (e.g. tumor thrombi). Knockdown of GATA2 expression enhanced migration and invasion of HepG2 cell in vitro (**Fig. 4C–D**). These results indicated that GATA2 may also be involved in the later stage of hepatocellular carcinoma, probably by inducing a more aggressive phenotype leading to distant metastasis as reported in prostate cancer [**27**]. Intratumoral GATA2 was differentially expressed in HCC tissues with recurrence or not and independently predicted patient outcome in univariate and multivariate analyses. These results highlighted the potential value of GATA2 as a new biomarker or target for HCC treatment.

GATA1 was thought as erythroid-expressed gene and restricted to the early stage of blood cell development [40]. Consistently, we showed complete loss of expression of GATA1 at both mRNA and protein levels in hepatic/HCC cell lines (**Fig. 1A and D**) and tissues (**Supplementary Fig. S1A**). GATA4 ∼ 6 are mainly expressed in organs of endoderm origin and involved in cellular proliferation and differentiation. Loss of function of GATA4 ∼ 6 has been correlated with progression of various solid tumors[14]–[16], [18]. Our results from immunohistochemistry indicated that majorities of HCC tissues exhibited cytoplasmic expression of GATA4 ∼ 6. This was in accordance with others’ reports showing loss of function of GATA4 ∼ 6 by aberrant nucleocytoplasmic localization [16], [17], which caused loss of expression of epithelial cell markers (Disabled-2, collagen IV, and laminin) and led to cellular dedifferentiation [16]. Our results also showed that expression of GATA4/5 was frequently lost while GATA6 was retained in majorities of hepatic/HCC cell lines at both mRNA and protein levels (**Fig. 1A and D**), which theoretically supported others’ result showing frequent hypermethylation of GATA4/5 rather than GATA6 in carcinoma cell lines and tissues [14], [18], [41]. GATA4 accounted for about half of the function of GATA families during hepatic development [19] and was considered to be one of the first transcription factors binding to chromatin during early endodermal differentiation, which has been proposed to initiate the opening of the chromatin and enable the binding of other transcription factors to DNA [39], [42]. A proper ratio of GATA4 to GATA6 function was important for the maintenance of chromatin structure as ‘differentiated’ or ‘dedifferentiated’ state. However, this didn’t seem to happen in hepatocellular carcinoma. Unlike the reciprocal expression pattern of GATA4 and GATA6 in adrenal tumors [43], expression of GATA4 and GATA6 positively correlated with each other at both mRNA and protein levels in tumor and peritumor tissues (**data not shown**). Designation of intratumoral GATA4-to-GATA6 ratio didn’t improve predictive value for TTR and OS compared with GATA4 or GATA6 alone (data not shown). Besides, well-differentiated HCC tissues had significantly increased expression of GATA2 (**Fig. 2** **, A2 vs A3**) and decreased expression of GATA4 (**Fig. 2** **, B2 vs B3**) proteins compared with poor-differentiated tissues. This evidence, together with others showing the inverse correlation of GATA4 with GATA6 in many cancers, drives us to assume that GATA4 is no longer the protagonist as it behaved in hepatic development due to frequent loss of expression in adult liver tumors [44]. We have demonstrated frequent loss of GATA4 and abundant expression of GATA2 in hepatic/HCC cell lines and tissues (**Fig. 1**). We therefore postulate that hematopoietic factor GATA2 has taken the place of endoderm factor GATA4 in hepatocarcinogenesis. Come up the question then whether GATA2 shares the same mechanism as GATA4 does in solid tumors or as it does in hematopoietic malignancies. Since information about regulation of GATA2 in solid tumors is still limited, further experiments are necessary.

In conclusion, for the first time, we evaluated the expression of GATA transcription factors and the prognostic potential for hepatocellular carcinoma after resection. Our results showed that GATA2 was differentially expressed in hepatic/HCC cell lines at both mRNA and protein levels and associated with pivotal clinicopathologic factors related to HCC invasiveness. Decreased expression of intratumoral GATA2 protein was demonstrated to be an independent prognostic factor for HCC outcome. RNAi-mediated knockdown of GATA2 significantly enhanced proliferation, migration and invasion of HepG2 cell in vitro. Our results exploit knowledge about GATA2 beyond its’ canonical role as hematopoietic factor. However, further experiments are needed to address how does liver gain ectopic expression of hematopoietic factor GATA2 with concomitant loss of endoderm factors GATA4 ∼ 6 and how, if that exists, do these factors cooperate or compete with each other in the progression of hepatocellular carcinoma.

## Supporting Information

Figure S1 **Correlation of average density with manual positive grade of GATAs protein.** GATA1 expression was negative in HCC (**A**). Average density results were highly agreed with manual positive grade method (negative, poor staining or strong staining) for GATAs protein (**B–E**). Mann-Whitney U tests.
(TIF)

Figure S2 **Kaplan-Meier analyses of GATA4 (A_1_–A_4_), GATA5 (B_1_–B_4_) and GATA6 (C_1_–C_4_) proteins for recurrence and death.** None of intratumoral or peritumoral GATA4 (**A_1_–A_4_**), GATA5 (**B_1_–B_4_**), GATA6 (**C_1_–C_4_**) expression showed prognostic value in terms of tumor recurrence or death. Frequencies of patients at risk, event or censored were created by life table method. High and low subgroups were defined by optimal cut-off using minimum p value approach.
(TIF)

Figure S3 **Kaplan-Meier analyses of intratumoral GATA2 in subgroups of patients receiving prophylactic therapy (C and D) or not (A and B) after resection.** In subgroups of patients receiving prophylactic therapy (**C–D**) or not (**A–B**), intratumoral GATA2 expression still showed prognostic value in terms of tumor recurrence or death. Frequencies of patients at risk, event or censored were created by life table method. High and low subgroups were defined by optimal cut-off using minimum p value approach.
(TIF)

Table S1 **Clinicopathologic Features of Patients.**
(DOC)

Table S2 **Primers for RT-qPCR.**
(DOC)

Table S3 **Correlation of GATA4,GATA5 and GATA6 Expression with Clinicopathologic Factors.**
(DOC)
