# Supplementary figures and images for: Decreased Expression of GATA2 Promoted Proliferation, Migration and Invasion of HepG2 In Vitro and Correlated with Poor Prognosis of Hepatocellular Carcinoma
Source: PLoS One. 2014 Jan 30;9(1):e87505. doi: 10.1371/journal.pone.0087505 (PMC3907524; doi:10.1371/journal.pone.0087505)

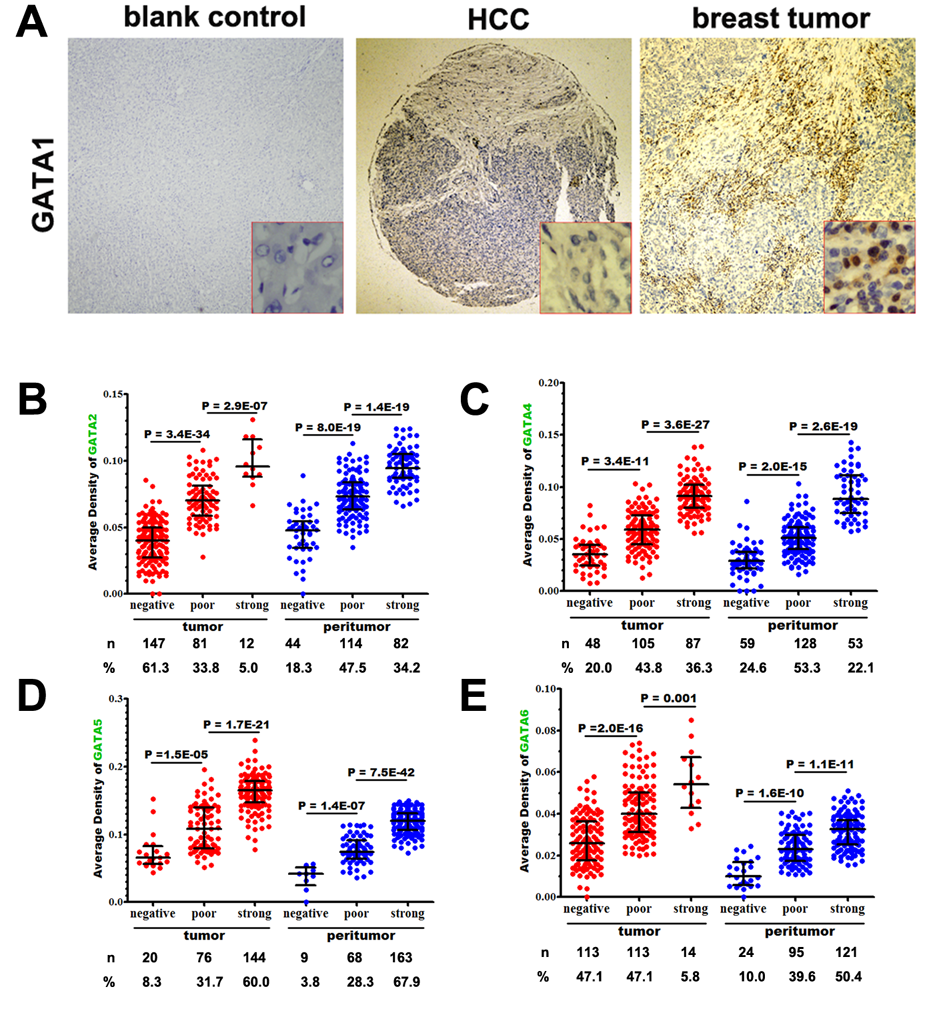

Supplement: Figure S1 — Correlation of average density with manual positive grade of GATAs protein. GATA1 expression was negative in HCC (A). Average density results were highly agreed with manual positive grade method (negative, poor staining or strong staining) for GATAs protein (B–E). Mann-Whitney U tests. (TIF) [file pone.0087505.s001.tif]

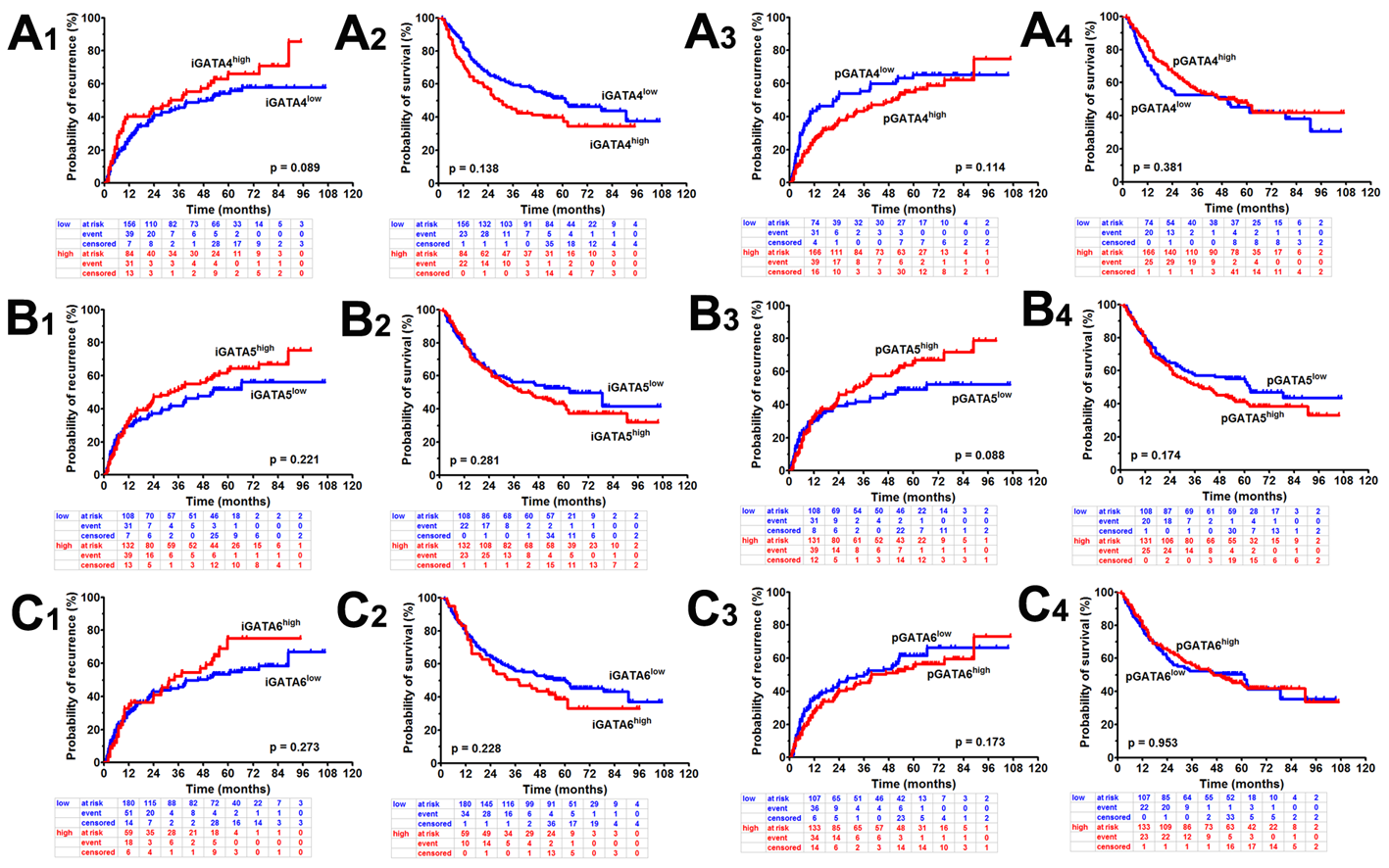

Supplement: Figure S2 — Kaplan-Meier analyses of GATA4 (A1–A4), GATA5 (B1–B4) and GATA6 (C1–C4) proteins for recurrence and death. None of intratumoral or peritumoral GATA4 (A1–A4), GATA5 (B1–B4), GATA6 (C1–C4) expression showed prognostic value in terms of tumor recurrence or death. Frequencies of patients at risk, event or censored were created by life table method. High and low subgroups were defined by optimal cut-off using minimum p value approach. (TIF) [file pone.0087505.s002.tif]

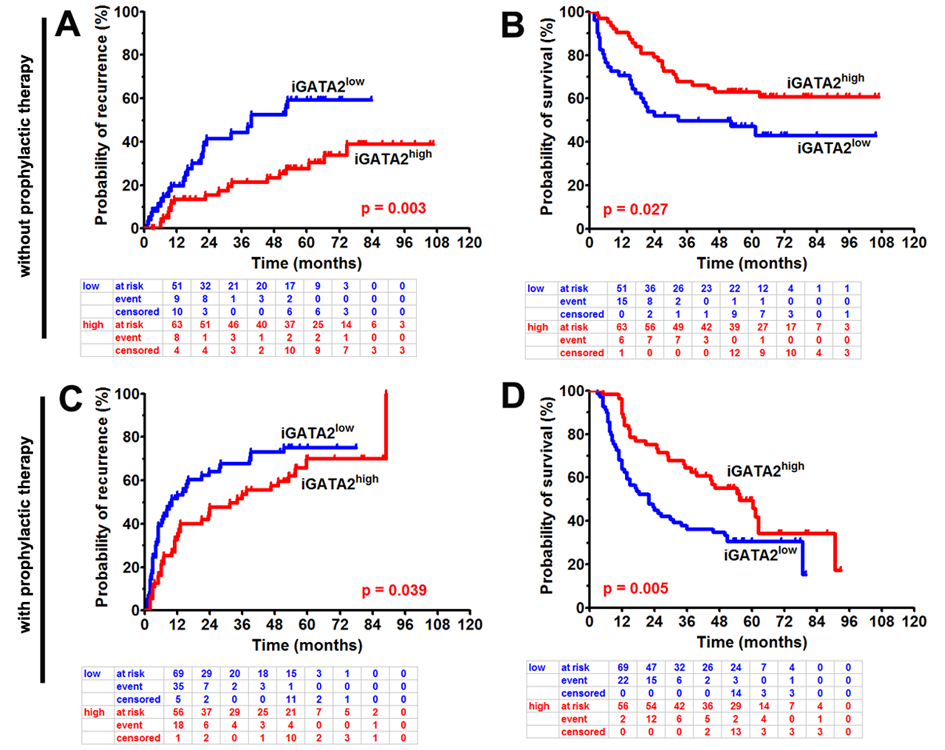

Supplement: Figure S3 — Kaplan-Meier analyses of intratumoral GATA2 in subgroups of patients receiving prophylactic therapy (C and D) or not (A and B) after resection. In subgroups of patients receiving prophylactic therapy (C–D) or not (A–B), intratumoral GATA2 expression still showed prognostic value in terms of tumor recurrence or death. Frequencies of patients at risk, event or censored were created by life table method. High and low subgroups were defined by optimal cut-off using minimum p value approach. (TIF) [file pone.0087505.s003.tif]
