# Supplementary material for: Decreased Expression of GATA2 Promoted Proliferation, Migration and Invasion of HepG2 In Vitro and Correlated with Poor Prognosis of Hepatocellular Carcinoma
Source: PLoS One. 2014 Jan 30;9(1):e87505. doi: 10.1371/journal.pone.0087505 (PMC3907524; doi:10.1371/journal.pone.0087505)
Supplement: Table S1 — Clinicopathologic Features of Patients. (DOC) [file pone.0087505.s004.doc]

**Supplementary Table S1**. Clinicopathologic Features of Patients

| Age, y, median (range) | 52 (18 ~ 81) |
| --- | --- |
| Gender (male/female) | 204/36 |
| Hepatitis infection (no/yes) | 16/224 |
| Liver cirrhosis (no/yes) | 28/212 |
| AFP, ng/ml, median (range) | 126.95 (0.85 ~ 60500) |
| γ-GT, U/L, median (range) | 64 (0 ~ 1111) |
| ALT, U/L, median (range) | 39 (6 ~ 1696) |
| Child-Pugh score (A/B) | 240/0 |
| Tumor size, cm, median (range) | 5.5 (0.9 ~ 23.0) |
| Tumor number (single/multiple) | 184/56 |
| Tumor capsule (yes/no) | 119/121 |
| Tumor differentiation (I/II/III/IV) | 7/128/102/3 |
| Tumor thrombi (no/microscopic/macroscopic/others)^a^ | 131/25/64/20 |
| TNM stage (I/II/III) | 106/76/58 |
| BCLC stage (0/A/B/C) | 23/30/78/109 |
| Prophylactic therapy (none/TACE/immunotherapy)^b^ | 114/124/2 |
| Post-recurrence therapy (none/TACE/regional/resection/others)^c^ | 40/54/4/21/7 |

**Abbreviations:** AFP, alpha-fetoprotein; γ-GT, gamma-glutamyl transpeptidase; ALT, alanine transaminase; TNM, tumor-node-metastasis; BCLC, Barcelona Clinic Liver Cancer; TACE, transcatheter arterial chemoembolization.

**a.** others: thrombi from hepatic vein, inferior caval vein or intrahepatic ducts.

**b.** immunotherapy: interferon-α or thymosin therapy.

**c.** regional: radio frequency ablation (RFA), percutaneous ethanol injection therapy (PEI) or microwave ablation(MA); others: traditional Chinese medicine etc.
