# Supplementary material for: Decreased Expression of GATA2 Promoted Proliferation, Migration and Invasion of HepG2 In Vitro and Correlated with Poor Prognosis of Hepatocellular Carcinoma
Source: PLoS One. 2014 Jan 30;9(1):e87505. doi: 10.1371/journal.pone.0087505 (PMC3907524; doi:10.1371/journal.pone.0087505)
Supplement: Table S2 — Primers for RT-qPCR. (DOC) [file pone.0087505.s005.doc]

**Supplementary Table S2**. Primers for RT-qPCR

| gene | sequence | Temperature (℃) | product (bp) |
| --- | --- | --- | --- |
| GATA1  NM_002049.3 | 5' GCCTCTATCACAAGATGAATGGG3'  5' CACAGTGTCGTGGTGGTCGT3' | 60 | 121 |
| GATA2  NM_001145661.1 | 5'CATCAAGCCCAAGCGAAGACT3'  5'CAGCTCCTCGAAGCACTCCG3' | 60 | 256 |
| GATA4  NM_002052.3 | F 5'CGGAAGCCCAAGAACCTGA 3'  R 5'GCTGCTGTGCCCGTAGTGA3' | 60 | 177 |
| GATA5  NM_080473.4 | 5'CGTCATCAATGCTCACCGTC 3'  5'GCACCACAGCTCCGTCTATC 3' | 60 | 203 |
| GATA6  NM_005257.3 | 5'CTCCAACTTCCACCTCTTCTAAC 3'  5'CCCATCTTGACCCGAATACTT 3' | 60 | 158 |
| ACTB  NM_001101.3 | F:5'TGGCACCCAGCACAATGAA3'  R:5'CTAAGTCATAGTCCGCCTAGAAGCA3' | 60 | 115 |
| HPRT1  NM_000194 | F:5'CCTGGCGTCGTGATTAGTG3'  R:5'CAGAGGGCTACAATGTGATGG3' | 60 | 182 |
