# Supplementary material for: Decreased Expression of GATA2 Promoted Proliferation, Migration and Invasion of HepG2 In Vitro and Correlated with Poor Prognosis of Hepatocellular Carcinoma
Source: PLoS One. 2014 Jan 30;9(1):e87505. doi: 10.1371/journal.pone.0087505 (PMC3907524; doi:10.1371/journal.pone.0087505)
Supplement: Table S3 — Correlation of GATA4,GATA5 and GATA6 Expression with Clinicopathologic Factors. (DOC) [file pone.0087505.s006.doc]

**Supplementary Table S3**. Correlation of GATA4,GATA5 and GATA6 Expression with Clinicopathologic Factors

|  |  | iGATA4 | | pGATA4 | | iGATA5 | | pGATA5 | | iGATA6 | | pGATA6 | |
| --- | --- | --- | --- | --- | --- | --- | --- | --- | --- | --- | --- | --- | --- |
|  |  | Mean Density | p | Mean Density | p | Mean Density | p | Mean Density | p | Mean Density | p | Mean Density | p |
| Gender | Male | 0.067±0.028 | 0.600 | 0.057±0.028 | 0.314 | 0.139±0.042 | 0.108 | 0.104±0.028 | 0.806 | 0.036±0.015 | 0.188 | 0.027±0.010 | 0.105 |
|  | Female | 0.064±0.027 |  | 0.052±0.026 |  | 0.130±0.041 |  | 0.103±0.031 |  | 0.033±0.015 |  | 0.024±0.009 |  |
| Age(Year) | ≦52 | 0.068±0.029 | 0.300 | 0.056±0.029 | 0.980 | 0.136±0.042 | 0.450 | 0.103±0.029 | 0.584 | 0.036±0.015 | 0.634 | 0.026±0.010 | 0.760 |
|  | >52 | 0.064±0.026 |  | 0.056±0.026 |  | 0.140±0.041 |  | 0.106±0.027 |  | 0.036±0.015 |  | 0.026±0.010 |  |
| HBV infection | No | 0.063±0.022 | 0.577 | 0.051±0.028 | 0.521 | 0.140±0.040 | 0.873 | 0.107±0.031 | 0.544 | 0.042±0.016 | 0.164 | 0.026±0.010 | 0.613 |
|  | Yes | 0.066±0.028 |  | 0.056±0.028 |  | 0.138±0.042 |  | 0.104±0.028 |  | 0.035±0.015 |  | 0.027±0.010 |  |
| Liver cirrhosis | No | 0.058±0.022 | 0.059 | 0.050±0.024 | 0.341 | 0.133±0.048 | 0.792 | 0.101±0.033 | 0.797 | 0.035±0.018 | 0.662 | 0.025±0.012 | 0.333 |
|  | Yes | 0.067±0.028 |  | 0.057±0.028 |  | 0.139±0.041 |  | 0.105±0.027 |  | 0.036±0.015 |  | 0.026±0.010 |  |
| AFP(ng/ml) | ≦20 | 0.060±0.026 | 0.014 | 0.053±0.027 | 0.209 | 0.137±0.043 | 0.994 | 0.104±0.029 | 0.957 | 0.035±0.016 | 0.722 | 0.026±0.010 | 0.976 |
|  | >20 | 0.069±0.028 |  | 0.058±0.028 |  | 0.138±0.041 |  | 0.104±0.028 |  | 0.036±0.015 |  | 0.027±0.010 |  |
| ALT(U/L) | ≦75 | 0.067±0.028 | 0.293 | 0.057±0.028 | 0.830 | 0.136±0.042 | 0.416 | 0.103±0.028 | 0.393 | 0.035±0.015 | 0.420 | 0.026±0.010 | 0.855 |
|  | >75 | 0.063±0.025 |  | 0.054±0.026 |  | 0.143±0.038 |  | 0.107±0.026 |  | 0.037±0.018 |  | 0.027±0.011 |  |
| γ-GT(U/L) | ≦54 | 0.065±0.028 | 0.604 | 0.053±0.026 | 0.296 | 0.138±0.042 | 0.863 | 0.106±0.028 | 0.260 | 0.037±0.014 | 0.357 | 0.027±0.010 | 0.307 |
|  | >54 | 0.067±0.027 |  | 0.058±0.029 |  | 0.138±0.042 |  | 0.103±0.028 |  | 0.035±0.016 |  | 0.026±0.010 |  |
| Tumor size(cm) | ≦5 | 0.068±0.028 | 0.220 | 0.057±0.026 | 0.488 | 0.139±0.041 | 0.974 | 0.104±0.026 | 0.996 | 0.036±0.014 | 0.937 | 0.027±0.010 | 0.156 |
|  | >5 | 0.064±0.027 |  | 0.055±0.029 |  | 0.137±0.042 |  | 0.104±0.030 |  | 0.036±0.166 |  | 0.026±0.010 |  |
| Tumor number | 1 | 0.066±0.027 | 0.961 | 0.056±0.027 | 0.849 | 0.140±0.040 | 0.211 | 0.105±0.027 | 0.360 | 0.036±0.015 | 0.207 | 0.027±0.010 | 0.799 |
|  | ≧2 | 0.066±0.029 |  | 0.057±0.031 |  | 0.130±0.043 |  | 0.100±0.031 |  | 0.034±0.172 |  | 0.026±0.010 |  |
| Tumor capsule | Yes | 0.064±0.027 | 0.144 | 0.057±0.028 | 0.727 | 0.140±0.040 | 0.524 | 0.107±0.027 | 0.227 | 0.037±0.014 | 0.085 | 0.027±0.010 | 0.859 |
|  | No | 0.068±0.028 |  | 0.056±0.028 |  | 0.135±0.043 |  | 0.102±0.029 |  | 0.034±0.016 |  | 0.026±0.010 |  |
| Differentiation | Well | 0.062±0.026 | 0.047 | 0.059±0.027 | 0.059 | 0.137±0.041 | 0.821 | 0.027±0.011 | 0.722 | 0.037±0.017 | 0.655 | 0.057±0.026 | 0.549 |
|  | Poor | 0.070±0.029 |  | 0.053±0.028 |  | 0.137±0.042 |  | 0.026±0.010 |  | 0.034±0.014 |  | 0.048±0.020 |  |
| Tumor thrombi | No | 0.064±0.026 | 0.143 | 0.056±0.026 | 0.813 | 0.135±0.042 | 0.094 | 0.105±0.028 | 0.904 | 0.036±0.015 | 0.948 | 0.027±0.010 | 0.386 |
|  | Yes | 0.069±0.029 |  | 0.057±0.029 |  | 0.142±0.041 |  | 0.103±0.028 |  | 0.036±0.016 |  | 0.026±0.010 |  |
| TNM stage | I | 0.062±0.026 | 0.069 | 0.056±0.028 | 0.857 | 0.137±0.041 | 0.501 | 0.106±0.027 | 0.468 | 0.037±0.014 | 0.343 | 0.027±0.010 | 0.271 |
|  | II/III | 0.069±0.028 |  | 0.056±0.028 |  | 0.139±0.042 |  | 0.103±0.028 |  | 0.035±0.017 |  | 0.026±0.010 |  |
| Prophylactic therapy | No | 0.068±0.026 | 0.246 | 0.055±0.027 | 0.681 | 0.138±0.041 | 0.757 | 0.105±0.029 | 0.227 | 0.036±0.015 | 0.793 | 0.026±0.010 | 0.787 |
|  | Yes | 0.064±0.028 |  | 0.055±0.028 |  | 0.138±0.041 |  | 0.101±0.028 |  | 0.035±0.015 |  | 0.026±0.009 |  |

Note: Mann-Whitney U tests for all the analyses.
